# Na,K-ATPase activity promotes macropinocytosis in colon cancer via Wnt signaling

**DOI:** 10.1242/bio.060269

**Published:** 2024-05-21

**Authors:** Nydia Tejeda-Muñoz, Yagmur Azbazdar, Eric A. Sosa, Julia Monka, Pu-Sheng Wei, Grace Binder, Kuo-Ching Mei, Yerbol Z. Kurmangaliyev, Edward M. De Robertis

**Affiliations:** ^1^ Department of Biological Chemistry, David Geffen School of Medicine, University of California, Los Angeles 90095-1662, USA; ^2^ Department of Oncology Science, University of Oklahoma Health Sciences Center, Oklahoma City, OK 73104, USA; ^3^ OU Health Stephenson Cancer Center, University of Oklahoma Health Sciences Center, Oklahoma City, OK 73104, USA; ^4^ Department of Genetics, Albert Einstein College of Medicine, Bronx, NY 10461, USA; ^5^ Department of Pharmaceutical Sciences, School of Pharmacy and Pharmaceutical Sciences, State University of New York at Binghamton, Binghamton, Johnson City, NY 13790, USA; ^6^ Department of Biology, Brandeis University, Waltham, MA 02453, USA

**Keywords:** Wnt signaling, Na, K-ATPase, Ouabain, Macropinocytosis, Colorectal carcinoma, Multivesicular bodies, *Xenopus laevis*

## Abstract

Recent research has shown that membrane trafficking plays an important role in canonical Wnt signaling through sequestration of the β-catenin destruction complex inside multivesicular bodies (MVBs) and lysosomes. In this study, we introduce Ouabain, an inhibitor of the Na,K-ATPase pump that establishes electric potentials across membranes, as a potent inhibitor of Wnt signaling. We find that Na,K-ATPase levels are elevated in advanced colon carcinoma, that this enzyme is elevated in cancer cells with constitutively activated Wnt pathway and is activated by GSK3 inhibitors that increase macropinocytosis. Ouabain blocks macropinocytosis, which is an essential step in Wnt signaling, probably explaining the strong effects of Ouabain on this pathway. In *Xenopus* embryos, brief Ouabain treatment at the 32-cell stage, critical for the earliest Wnt signal in development-inhibited brains, could be reversed by treatment with Lithium chloride, a Wnt mimic. Inhibiting membrane trafficking may provide a way of targeting Wnt-driven cancers.

## Teaser

**Summary:** Membrane trafficking's pivotal role in Wnt signaling unveiled; Ouabain inhibits the Wnt pathway, offering a potential strategy against Wnt-driven cancers.

## INTRODUCTION

The sodium–potassium ATPase pump (Na,K-ATPase) is a ubiquitously expressed transmembrane protein essential for maintaining the Na^+^ and K^+^ gradient that generates an electrical resting potential across the cell membrane by transporting three Na+ ions outside and two K+ ions inside the cell against their concentration gradients by hydrolyzing ATP (Lingrel, 2010). Ouabain is a specific inhibitor of the alpha subunit of this key membrane enzyme. Ouabain/digitalis steroid compounds have a long history in the medical treatment of cardiac insufficiency and arrhythmias (Lingrel, 2010). In addition to its ion transport function, Na,K-ATPase has cell signaling activity. When Ouabain binds to Na,K-ATPase, the Src Tyrosine kinase in caveolae is activated (Pierre and Xie, 2006), which could be a target for developing anticancer drugs due to its implications in cell adhesion, and progression of cancer subtypes. Multiple reports show that targeting Na,K-ATPase inhibits the proliferation of tumor cells (Barwe et al., 2005), highlighting the importance of Na,K-ATPase as a therapeutic target in the cancer field. In recent years it has become increasingly evident that cellular signaling pathways do not operate in isolation; rather, they are interconnected and communicate with each other to orchestrate a variety of critical biological processes. An intriguing area of research investigating the interaction between the Na,K-ATPase enzyme, and the Wnt signaling pathway is emerging.

The Wnt signaling pathway is one of the most ancient and well-studied pathways that regulates embryonic development, influencing processes such as axis formation, cell proliferation and differentiation, cell fate decisions, and brain wiring (Clevers et al., 2014; Kurmangaliyev et al., 2019; Sarin et al., 2018). Wnt signaling drives a cellular growth program that can become inappropriately activated in cancer. Sequestration of the serine/threonine protein kinase GSK3 (Glycogen Synthase Kinase 3β) is vital in activating the canonical Wnt pathway (Taelman et al., 2010). When the Wnt ligands bind to the Frizzled (Fz) receptor and the Lrp6 co-receptor, GSK3 is translocated into the membrane. It is then internalized into an early endosome and multivesicular bodies (MVBs). The sequestration of GSK3 and the destruction complex activate the Wnt pathway (Taelman et al., 2010). It was found that in macropinocytic cells, internalization of the plasma membrane is required for sustained Wnt signaling (Redelman-Sidi et al., 2018; Tejeda-Muñoz and De Robertis, 2022) and subsequent rapid metabolic changes (Albrecht et al., 2020; Tejeda-Muñoz et al., 2019).

Recent research has revealed a reciprocal interaction between the Na,K-ATPase pump and the Wnt pathway, suggesting that these two seemingly distinct pathways may mutually influence each other to modulate a cellular response (Sarin et al., 2018; Taelman et al., 2010; Albrecht et al., 2020). At a molecular level, it has been reported that Ouabain modulates the activation of important signaling pathways in synapse formation, such as CREB-BDNF, NFκB, GSK-3β, and Wnt/β-Catenin (Orellana et al., 2018). Furthermore, the Na,K-ATPase pump has similarly been implicated in regulating GSK-3β (Sopjani et al., 2010). The molecular interactions between the Na,K-ATPase and the Wnt pathway have significant functional implications in cellular regulation and disease development. Modulating Na,K-ATPase activity may influence Wnt pathway activity and biological processes (Huang et al., 2022). This interconnection suggests potential therapeutic strategies that could harness these interactions to address diseases associated with aberrant Wnt pathway activation, such as cancer.

In the present study, we investigated whether macropinocytosis is involved in regulating the Na,K-ATPase pump via Wnt signaling. We report that the Na,K-ATPase is macropinocytosed and internalized in lysosomes after activation of the Wnt pathway. The selective Na,K-ATPase inhibitor Ouabain blocked Wnt-induced internalization of Tetramethylrhodamine-dextran with a hydrated diameter of over 200 nm (TMR-dextran), as well as β-catenin accumulation and transcriptional signaling in cultured cells, including colon cancer cells in which the Wnt pathway is constitutively activated. Using the *Xenopus* embryo model system, we found that inhibiting Na,K-ATPase in embryos blocked the effect of the GSK3 inhibitor Lithium chloride (LiCl) and Wnt. Treatment of *Xenopus* embryos for a brief period with Ouabain at the 32-cell stage inhibited the early Wnt signal, resulting in ventralized embryos lacking head structures. The relationship between the Na,K-ATPase, and the Wnt signaling pathway at the level of membrane trafficking represents an exciting new field of research. Understanding the underlying molecular mechanisms and functional implications of this interaction holds the potential to further understand developmental processes as well as implement novel therapeutic strategies in cancer.

## RESULTS

### Na,K-ATPase is overexpressed in colon cancer and correlates with cancer progression

**Fig. 1.**
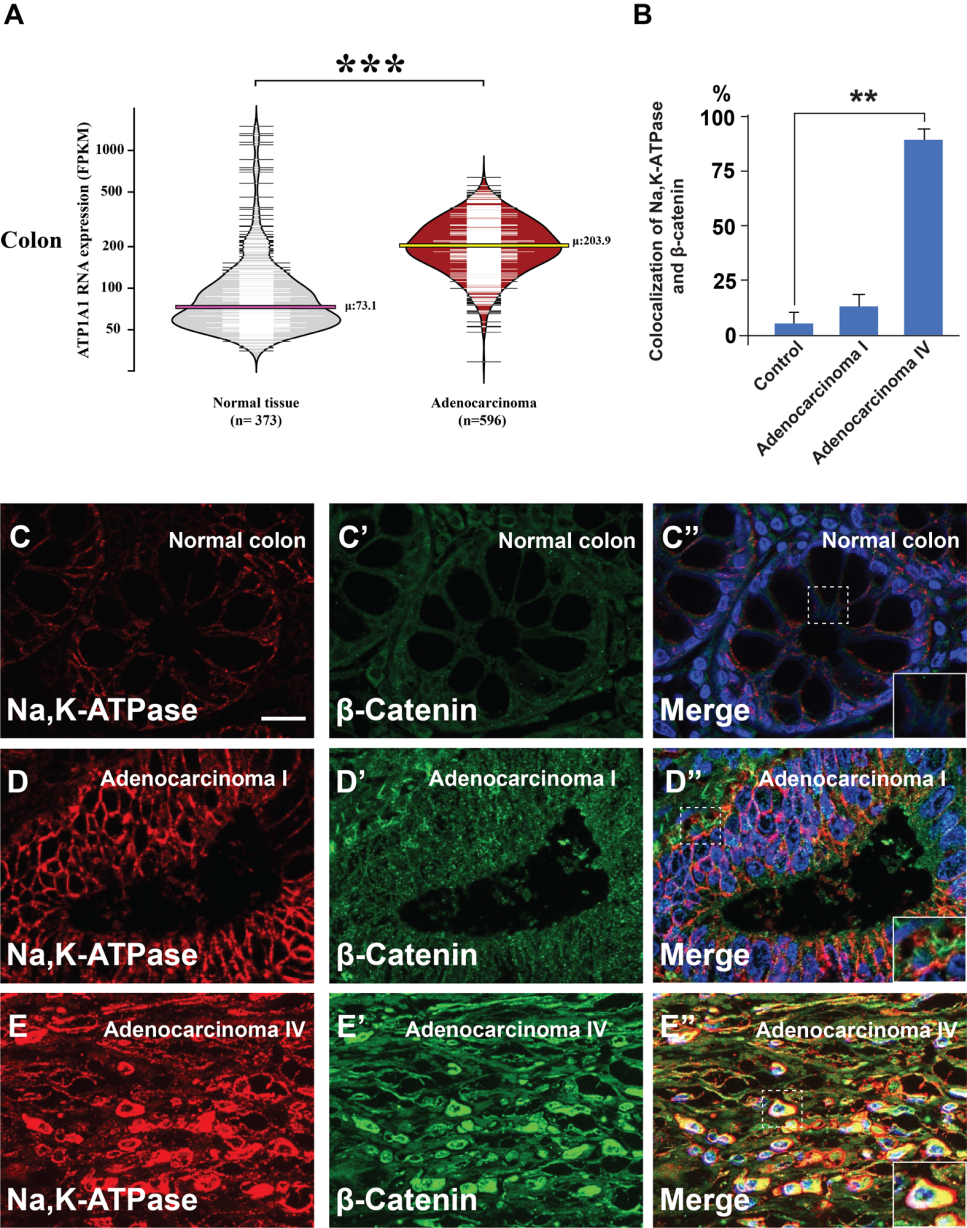
**Na,K-ATPase subunit α is overexpressed in colon cancer.** (A) ATP1A1 RNA overexpression is significantly associated with colon adenocarcinoma. Bean plot analyses of ATP1A1 expression in 596 adenocarcinomas and 373 healthy colon samples reveal an approximately threefold difference in expression. The width of the horizontal lines represents the number of samples at each value, while the pink and yellow lines indicate the average ATP1A1 expression in normal colon and adenocarcinoma, respectively. The triple black asterisk indicates a *P*-value of 2.33×10^−23^ obtained via a two-tailed Student's *t*-test. (B) Quantification of the colocalization between Na,K-ATPase, and β-catenin in normal colon and in advanced stages of cancer from human array sections. (C-E″) Immunohistochemistry from B shows strong colocalization between Na,K-ATPase and β-catenin in advanced colon cancers (inset). Error bars denote SEM (*n*≥3) (**P<0.01). Scale bars: 10 μm. Human array sections were quantified in triplicate fields.

The Na,K-ATPase has been implicated in membrane potential, signal transduction, and transcriptional activation (Wu et al., 2013), regulating cellular proliferation, motility, and apoptosis (Aperia, 2007). To investigate the role of the Na,K-ATPase α1 subunit (ATP1A1), which is the subunit that binds to Ouabain, we examined its expression in healthy colon tissue and adenocarcinoma by using its expression data available in the Human Protein Atlas (Thul and Lindskog, 2018). ATP1A1 mRNA expression was analyzed in 373 normal colon tissues and 596 adenocarcinoma samples. Bean plot analysis revealed that the average ATP1A1 expression in normal tissue was 73.1 FPKM (horizontal pink line), and expression levels increased to an average of 203.9 FPKM in colon adenocarcinoma (horizontal yellow line) (Fig. 1B). This difference in ATP1A1 expression was statistically significant via two-tailed Student's *t*-test (*P*=2.33×10^−23^). This finding was confirmed immunohistochemically in colonic cancer tissue arrays [containing 90 cases of adenocarcinoma of various grades I-IV and 90 samples of corresponding adjacent normal tissues (from TissueArray)] which were immunostained for β-catenin and Na,K-ATPase antibodies (Fig. 1C-E″). Using β-catenin as a control for Wnt signaling activation, we found that Na,K-ATPase protein levels strongly correlated with advanced grade IV malignancy when compared to the normal colon. These *in vivo* observations of human tumors were consistent with the view that membrane trafficking promotes colon cancer progression.

### Na,K-ATPase is regulated by the canonical Wnt signaling

It was reported that targeting Na,K-ATPase can affect cell death, highlighting its role in cancer research (Litan and Langhans, 2015). The Na,K-ATPase is expressed in different cancers such as glioblastoma (Mijatovic et al., 2008), melanoma (Mathieu et al., 2009), and non-small cell lung cancer (NSCLC). Importantly, the α3 isoform has been reported to be overexpressed in colon cancer (Baker Bechmann et al., 2016). The canonical Wnt/β-catenin signaling pathway is the most common signaling pathway driving colon cancer (Kinzler and Vogelstein, 1996; Clevers and Nusse, 2012). Recent reports show that the Wnt pathway also has an important role in membrane trafficking and cell-matrix adhesion (Mijatovic et al., 2008; Mathieu et al., 2009).

**Fig. 2.**
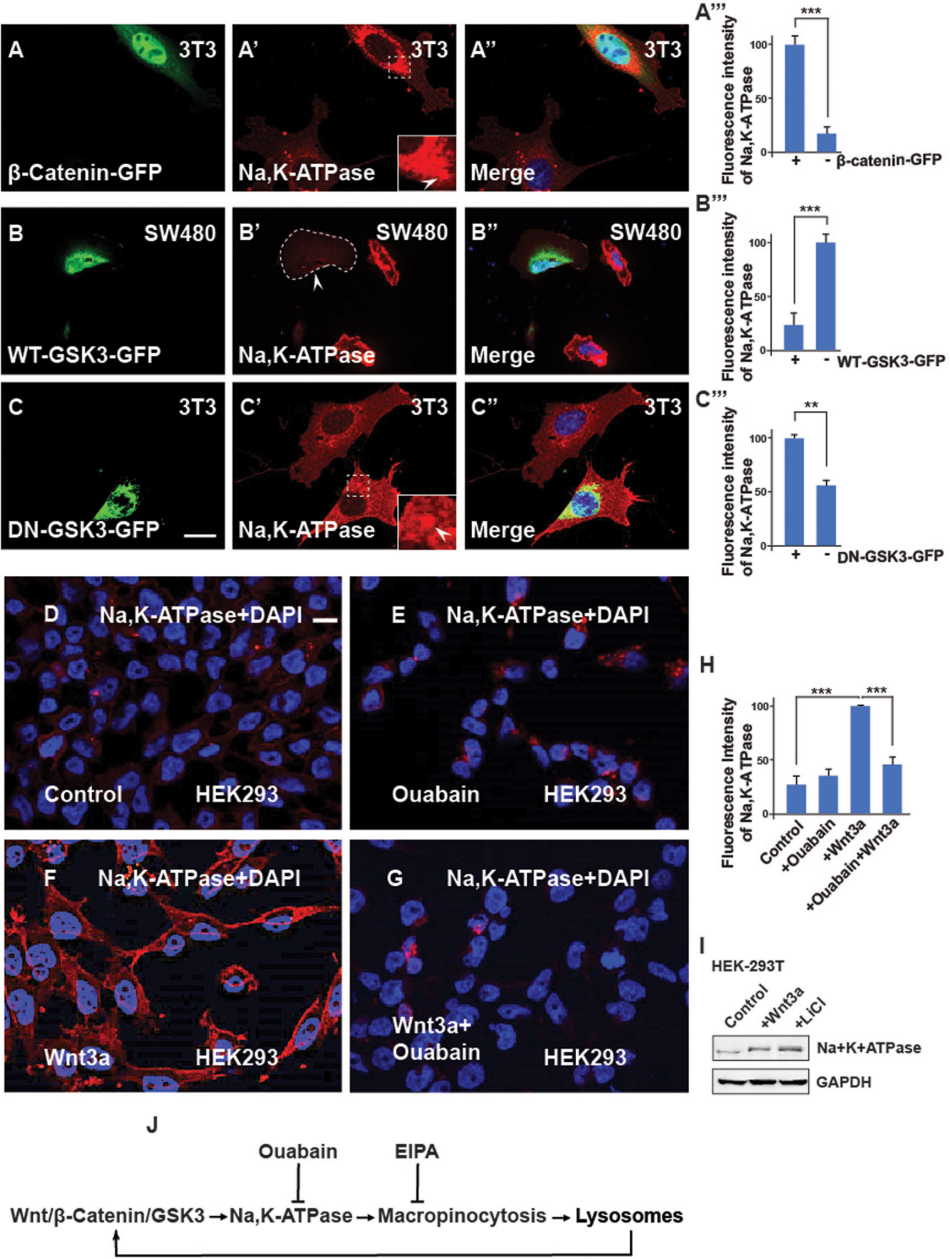
**The Na,K-ATPase is required for Wnt signaling and its regulation is via GSK3.** (A-A‴) 3T3 cells transfected with the stabilized constitutively active forms of β-catenin-GFP show increased levels of Na,K-ATPase compared to untransfected neighbor cells used as a control. Quantification is shown on the right side. (B-B‴) Overexpression of WT-GSK3-GFP (demarcated by a stippled line) in CRC SW480 cells blocks the Na,K-ATPase stabilization by Wnt. (C-C‴) Transfecting DN-GSK3-GFP (DN-GSK3-GFP) in 3T3 cells increases Na,K-ATPase levels. Note that the accumulation is observed in vesicles (indicated by the stippled box). (D) HEK293BR (BAR/Renilla) cells stained with Na,K-ATPase antibody. (E) HEK293BR cells treated with the Na,K-ATPase inhibitor Ouabain (1 µM) overnight showed no changes in Na,K-ATPase levels. (F) Overnight treatment with Wnt3a protein (100 ng/ml from Peprotech) treatment stabilized Na,K-ATPase. (G) Ouabain blocked Na,K-ATPase stabilization due to Wnt3a. (H) Quantification of the fluorescence intensity of the Na,K-ATPase from D-G. (I) Western blot showing that endogenous levels of Na,K-ATPase increase after activating the canonical Wnt pathway. (J) Diagram showing that Na,K-ATPase is positively regulated by Wnt via macropinocytosis in lysosomes. EIPA blocks macropinocytosis; Ouabain blocks the Na,K-ATPase, and lysosomes enhance Wnt. All experiments with cultured cells were biological triplicates. Scale bars: 10 μm. Error bars denote s.e.m. (*n*≥3) (**P<0.01, ***P<0.001).

To investigate the role of Na,K-ATPase in Wnt signaling, 3T3 cells were transfected with a Wnt pathway activator (stabilized β-catenin-GFP) (Barwe et al., 2005), and stained with Na,K-ATPase antibody. Cells with activated Wnt signaling were found to have elevated intracellular levels of Na,K-ATPase expression (Fig. 2A, compare transfected cell with untransfected cell). In addition, transfection of wild-type GSK3-GFP decreased (Fig. 2B, arrowhead) and catalytically inactive dominant-negative GSK3-GFP increased Na,K-ATPase staining (Fig. 2C) Similar results showing stabilization of Na,K-ATPase were observed after Wnt3a protein (100 ng/ml from Peprotech, overnight) treatment (Fig. 2D, compare with panel 2G). Interestingly, Ouabain treatment eliminated the increase in Na,K-ATPase levels induced by Wnt3a protein treatment (Fig. 2D-H).

Since Na,K-ATPase was accumulated in regions of potential lysosomal localization, we used SW480 colon cancer cells with constitutive Wnt signaling resulting from a mutation of adenopolyposis coli (APC) (Faux et al., 2004) that had elevated levels of lysosomes (Clevers et al., 2014). SW480 cells co-localized with the CD63 MVB/Lysosome marker, indicating strong localization (Fig. S1). This suggests that Na,K-ATPase accumulates and is stabilized in lysosomes in SW480 cells with APC mutation and HEK293 cells with Wnt activation (Fig. S1). It was recently reported that macropinocytosis is required for early Wnt signaling and head development in the *Xenopus in vivo* model (Kurmangaliyev et al., 2019). To investigate whether macropinocytosis is also involved in the regulation of the Na,K-ATPase, SW480 cells were treated with the Na^+^/H^+^ exchanger inhibitor ethylisopropylamiloride (EIPA) that blocks macropinocytosis (Koivusalo et al., 2010), which resulted in a reduction of the levels of the lysosomal marker CD63 and Na,K-ATPase, (Fig. S1B-B″ and S1E-E″) in SW480 cancer cells.

We next investigated the role of Ouabain, a ligand of Na,K-ATPase that inhibits ATP-dependent sodium–potassium exchange across cell membranes. We found that similarly to EIPA, Ouabain also reduced Na,K-ATPase, CD63, and β-catenin levels (Fig. S1C-C″ and S1E-E″), as well as Na,K-ATPase with β-catenin (F-F″). In HEK293 cells, Ouabain also inhibited CD63 levels, even when Wnt signaling and MVB/lysosome accumulation were stimulated with the GSK3 inhibitors LiCl or Chiron 99021 (Fig. S1G-M).

Taken together, the results suggest a possible pathway in which Na,K-ATPase may positively regulate Wnt signaling, macropinocytosis and lysosomes, as indicated in Fig. 2H. Expression of macropinocytosis components, MVBs, and lysosomes increased with colorectal cancer (CRC) malignancy, which can be targeted with Ouabain.

### Macropinocytosis induced by GSK3 inhibition is blocked by Ouabain

**Fig. 3.**
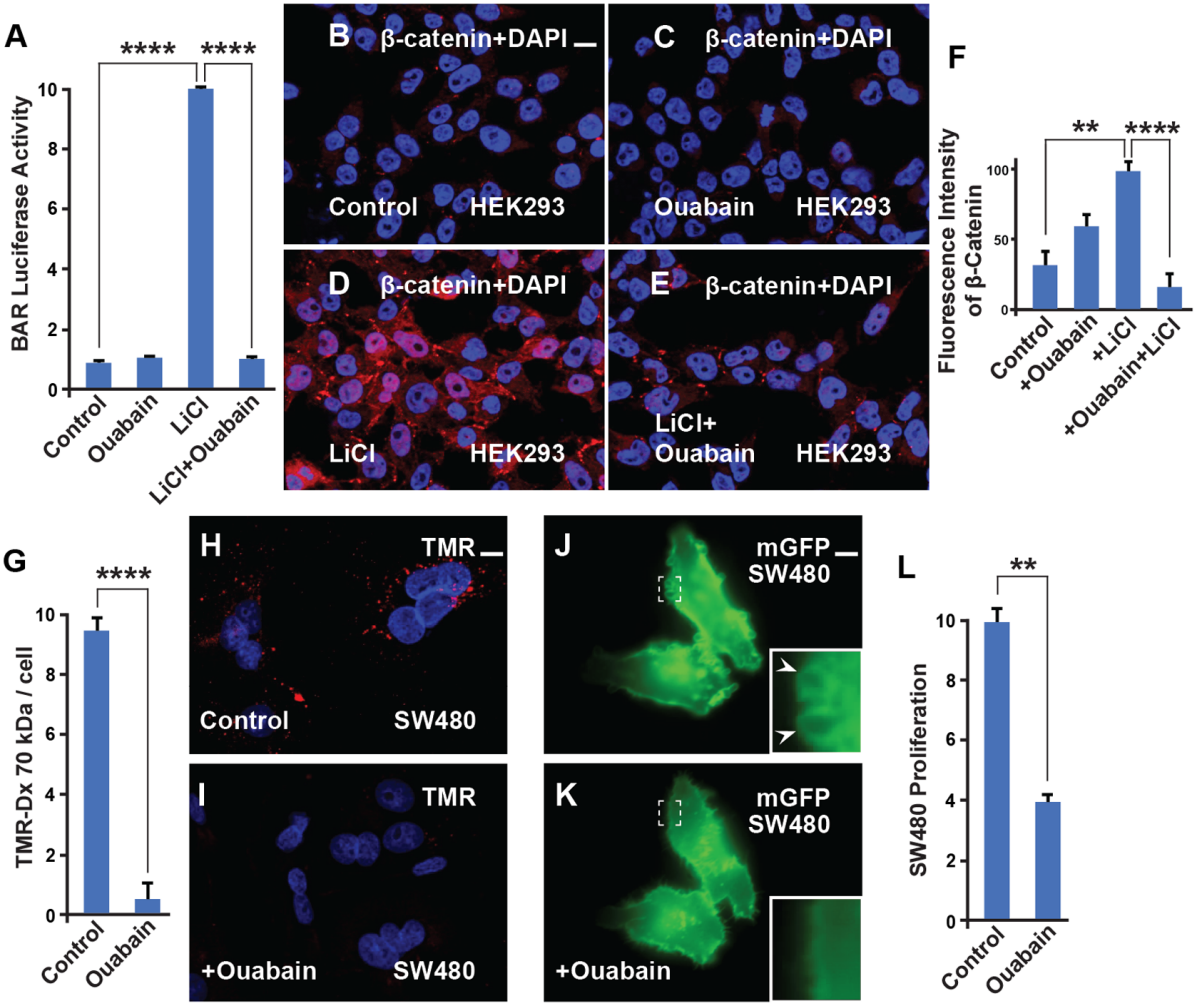
**Ouabain inhibits β-catenin signaling, macropinocytosis, and cell proliferation.** (A) β-catenin transcriptional activity stimulated by Wnt3a was strongly inhibited by Ouabain in HEK293BR cells. (B) Untreated HEK293BR (BAR/Renilla) cells stained with total β-catenin antibody. (C) HEK293BR (BAR/Renilla) cells treated with Ouabain showed no changes in β-catenin levels. (D) LiCl (40 mM) activates the β-catenin transcriptional response as shown in the BAR-luciferase assay. This effect was blocked with Ouabain (1 µM) treatment. (E) The increase of β-catenin levels due to LiCl, which increases macropinocytosis (Albrecht et al., 2020), was blocked by Ouabain. (F) Quantification of the β-catenin levels from B and E. (G) Quantification of the macropinocytosis uptake of TMR-dextran 70 kDa into SW480 cells and its reduction by Ouabain treatment. (H) TMR-dextran 1 (1 mg/ml) uptake into SW480 cells after 1 h of incubation. (I) TMR-dextran uptake after 1 h of incubation is reduced with Ouabain (200 nM) treatment. (J-K) SW480 cells transfected with membrane-GFP display sustained macropinocytic vesicles (arrowhead and inset) that were rapidly blocked within 30 min of Ouabain treatment (still images from Movie 1). (L) Cell proliferation in SW480 cells is decreased by Ouabain treatment (200 nM), only viable cells were scored. All experiments with cultured cells were biological triplicates. Error bars denote SEM (*n*≥3) (**P<0.01, ****P<0.0001). Scale bar: 10 μm.

LiCl mimics Wnt signaling through the inhibition of GSK3. To investigate whether Ouabain works at the transcriptional level and blocks the effect of LiCl, stably transfected HEK293 BAR-Luciferase/Renilla cells (HEK294BR) (Clevers et al., 2014) were incubated with LiCl in the presence or absence of Ouabain. The results revealed a strong inhibition of Wnt signaling (Fig. 3A-F). This suggests that Ouabain blocks the effect of Na,K-ATPase and works at the level of GSK3 and the Wnt pathway.

We also tested a different Na,K-ATPase inhibitor. It has been reported that extensive macropinocytosis begins very early in infection in human respiratory syncytial virus (RSV) infected cells (Baker Bechmann et al., 2016). Macropinosome formation under these conditions was shown to be dependent upon ATP1A1 and was significantly reduced if ATP1A1 expression was decreased or if the cells were treated with Ouabain or Rostafuroxin (also known as PST2238). The Na,K-ATPase inhibitor Rostafuroxin binds specifically to the ATP1A1 extracellular domain and blocks RSV-triggered EGFR Tyr845 phosphorylation. Given this connection between Rostafuroxin-macropinocytosis and ATP1A1 (Lingemann et al., 2019), we used this as an additional strategy to confirm that Na,K-ATPase was affecting the Wnt pathway independently of the Ouabain binding site. It was found that Rostafuroxin/PST2238 inhibited Wnt3a signaling in a β-catenin transcriptional assay (Fig. S2A). To corroborate the requirement of Na,K-ATPase for Wnt signaling, a BAR (β-catenin-activated luciferase reporter) assay with ATP1A1 siRNA was conducted. This assay showed inhibition of Wnt signaling when compared with the siRNA scrambled control, particularly evident in SW480 (Fig. S2B, lane 2). Quantification by flow cytometry shows that overexpressing the ATP1A1 enhances the activation of the Wnt pathway by LiCl (Fig. S2C).

The SW480 colon adenocarcinoma cell model system, frequently used to study the molecular mechanisms of colorectal cancer, has constitutive macropinocytosis and Wnt signaling. To test whether Ouabain affects macropinocytosis triggered by Wnt activation TMR-dextran was used in SW480 cells. The results showed that Ouabain blocked TMR macropinocytosis in SW480 cancer cells, (Fig. 3G-L; Fig. S2), decreased macropinosome plasma membrane formation within minutes of addition (Fig. 3J-K; Fig. S3E-G, and Movie 1), and reduced cell proliferation (Fig. 3L; Fig. S1), suggesting a possible role as a novel therapeutic agent for Wnt-driven cancers.

### The Na,K-ATPase is required for dorsal signaling activation in *Xenopus*

**Fig. 4.**
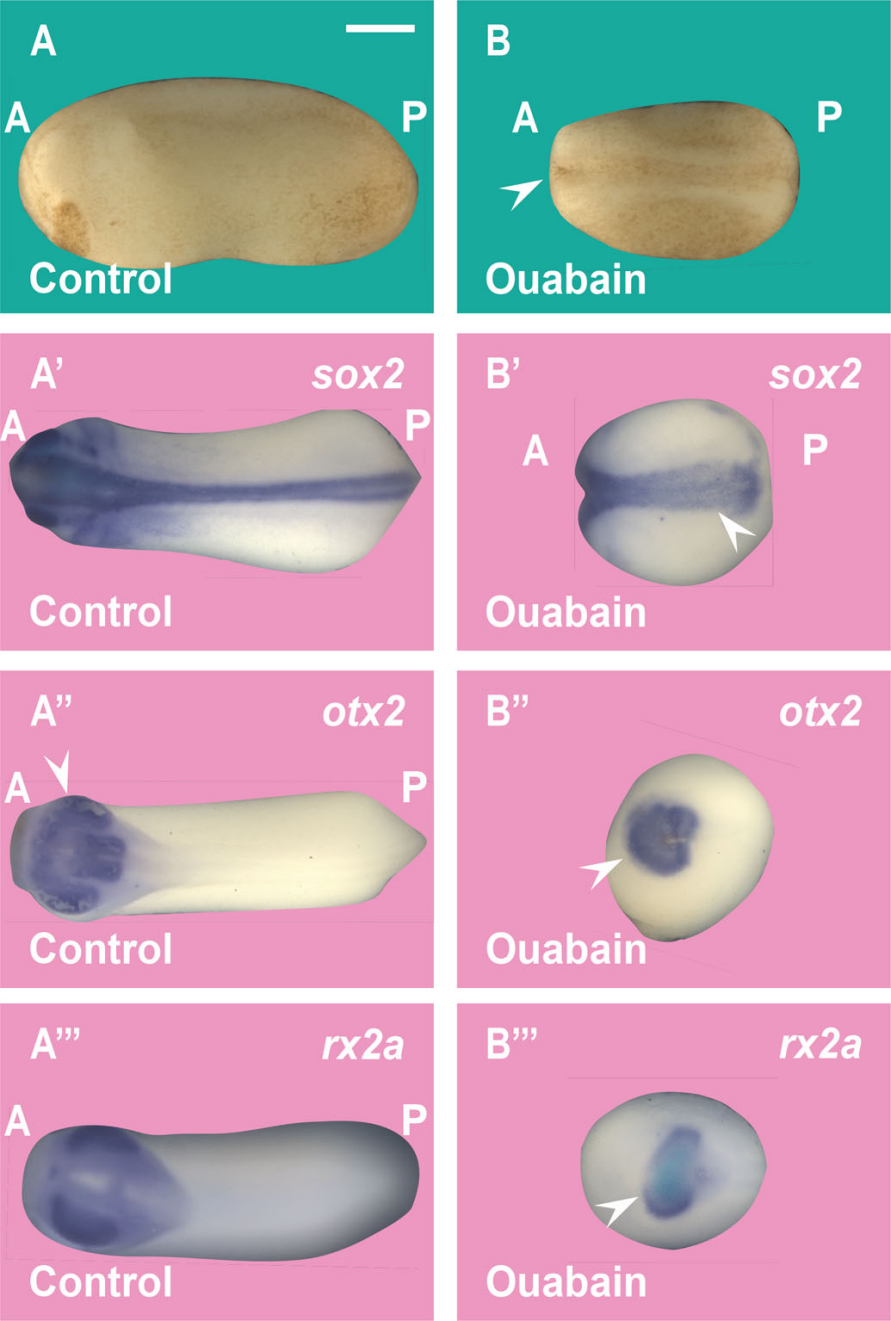
**Ouabain inhibits anterior CNS development in *Xenopus* embryos.** (A-B) Incubation of *Xenopus* embryos at the 32-cell stage in 10 μM Ouabain for 7 min results in embryos with a ventralized (small head) phenotype. A, anterior; P, posterior. (A′-B′) Ouabain incubation reduced the pan-neural marker *sox2* in *Xenopus* embryos. (A″-B″) Ouabain incubation reduces the forebrain and midbrain marker *Otx2* in *Xenopus* embryos (arrowhead). (A‴-B‴) Ouabain incubation reduces the eye marker *Rx2a* in *Xenopus* embryos. The numbers of embryos analyzed were as follows: A: 130, 100%; A′: 138, 97%; five independent experiments; A′: 32, 100%; B′: 36; 97%; A″: 32, 100%; B″: 28, 92%; A‴: 30, 100%; B‴: 27, 93%. Scale bar: 500 μm.

Na,K-ATPase has been implicated in multiple developmental processes (Kinzler and Vogelstein, 1996; Clevers and Nusse, 2012; Tejeda-Muñoz and De Robertis, 2022a). We examined the effects of Na,K-ATPase inhibition on the activity of β-catenin activation using the double-axis formation assay in *Xenopus* embryos (Fig. 4). It is known that the peak of maximal dorsalization by LiCl treatment is at the 32-cell stage (Kao et al., 1986), while inhibiting lysosomes with the V-ATPase inhibitors Baf (Bafilomycin A) or Con (Concanamycin A) ventralized embryos at the same developmental stage (Mijatovic et al., 2008). When embryos were incubated with the Na,K-ATPase inhibitor Ouabain for 7 min at the 32-cell stage, dorso-anterior development was reduced (Fig. 4A′-B‴). Using *in situ* hybridization with the pan-neural marker *Sox2*, the forebrain-midbrain marker *Otx2*, and the eye marker *Rx2a* it was found that anterior central nervous system (CNS) development requires Na,K-ATPase activity during the 32-cell stage (Fig. 4A′-B‴). This finding suggests that Na,K-ATPase enzyme activity is required for the initiation of the endogenous dorsal early Wnt signaling pathway in *Xenopus*.

### The 32-cell stage is critical for dorsal–ventral pattern decisions mediated by GSK3

**Fig. 5.**
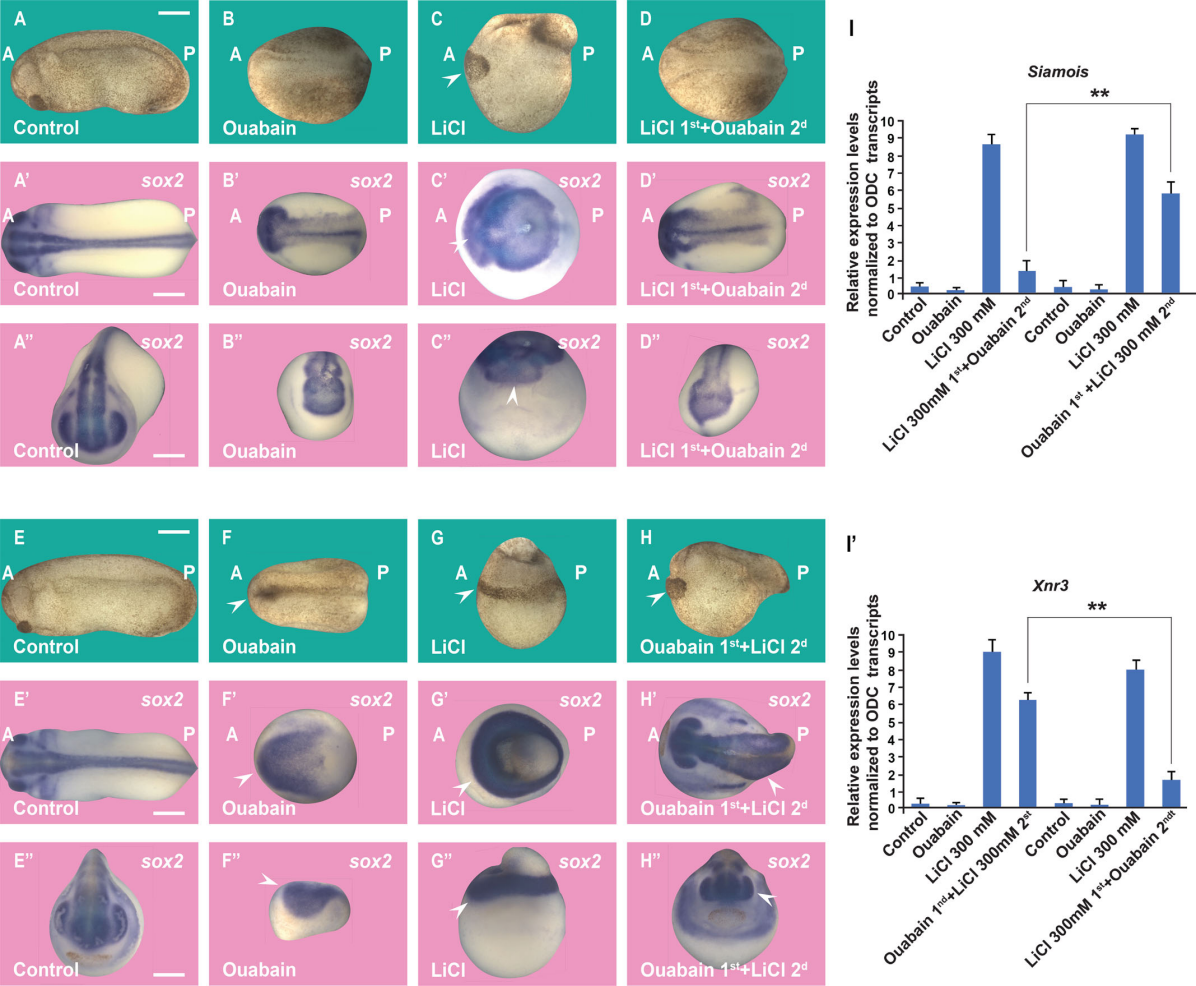
**Order-of-addition experiment showing that Wnt/β-catenin activation at the 32-cell stage is inhibited by Ouabain and can be rescued by subsequent GSK3 inhibition in *Xenopus* embryos.** (A) Untreated embryo. (B) Ouabain incubation (10 μM in 20% L-15 medium for 7 min at the 32-cell stage) resulted in ventralized embryos. (C) LiCl (300 mM for 7 min) dorsalized embryos resulting in expanded heads and reduced trunks. (D) The dorsalized phenotype due to LiCl treatment was blocked after subsequent incubation with Ouabain. (A′-D′) *In situ* hybridization of *Sox2* expression at the gastrula stage showing reduction of CNS development by Ouabain and its expansion by LiCL in dorsal views. The radial expanded CNS phenotype by treating first with LiCl incubation was inhibited by secondary Ouabain incubation. (A″-D″) Anterior views of the same embryos shown in A′-D′. (E-H) Embryos at the 32-cell stage were treated with Ouabain for 7 min (10 µM in 20% L-15 culture medium without serum), washed twice in 0.1 MMR saline, then immersed in 300 mM LiCl in MMR for an additional 7 min, all within the 32-cell stage. Note that treatment with LiCL secondly restored head structures. (E′-H′) *In situ* hybridizations of the neural marker *Sox2* at the gastrula stage show that the ventralized phenotype due to Ouabain was rescued by subsequent LiCl treatment. (E″-H″) Anterior views from the same embryos are shown in panels E′ to H′. (I-I′) Quantitative RT-PCR (qPCR) for the Wnt target genes *Siamois* and *Xnr3* at blastula stage 9 (about 7 h of development), shows that the phenotypic effects are due to the early activation of the Wnt pathway. The numbers of embryos analyzed are as follows: A: 82, 100%; B: 95, 96%; C: 110, 99%; D: 87, 94%; five independent experiments; A′: 33, 100%; B′: 29, 92%; C: 34, 100%; D: 31, 92%; E: 78, 100%; F: 83, 95%; G: 92, 99%; H: 85, 94%; E′: 28, 100%; F′: 33, 96%; G′: 30, 100%; H′: 35, 95%. Scale bars: 500 μm. Error bars denote SEM (*n*≥3) (**P<0.01).

LiCl treatment during the sensitive 32-cell stage in *Xenopus* generates truncated tadpoles with enlarged dorsal-anterior structures and reduced trunk structures (Fig. 5A,C), while a 7-min incubation at the 32-cell stage with Ouabain ventralized the embryos (Fig. 5B). To test that Ouabain works in the early Wnt pathway during development, we carried out-of-order addition experiments. When embryos were incubated with LiCl first, washed, and subsequently briefly treated with Ouabain, it was found that the expanded head lacking trunk structures phenotype due to LiCl treatment was blocked with Ouabain (Fig. 5D). The CNS *Sox2* marker confirmed that the large brain phenotype was blocked (Fig. 5A′-D″). Using qRT-PCR of the Wnt direct transcriptional targets *Siamois* and *Xnr3* at the blastula stage, we showed that Ouabain inhibits the Wnt pathway (Fig. 5I-I’). The converse experiment, a primary incubation with Ouabain, wash, and secondary incubation with LiCl, rescued the CNS deficiency phenotype (Fig. 5E-H″) These results were confirmed by qRT-PCR results using *Siamois* and *Xnr3* at late blastula as Wnt reporters (Fig. 5I-I’). We conclude that Ouabain blocks the early Wnt/β-catenin/GSK3 pathway signal in *Xenopus* embryos.

## DISCUSSION

In the present study, we report that inhibition of the multifunctional enzyme Na,K-ATPase is required for Wnt signaling pathway in cultured cells, cancer, and early development. Targeting Na,K-ATPase activity with an Ouabain inhibitor, which targets the α1 subunit, strongly reduced proliferation, macropinocytosis, and Wnt signaling in several cell lines. Na,K-ATPase has become an attractive target for cancer therapy because of its aberrant expression and activity in different types of human cancer. However, the dysregulation of particular Na,K-ATPase subunits varies among cancers; thus, its role in tumor development is still unclear. It is known that the α1 subunit of the Na,K-ATPase (ATP1A1) is overexpressed in non-small-cell lung cancer, renal clear cell carcinoma, glioblastoma, melanoma and, as shown here, colon cancer. We found that with Wnt/β-catenin activation, the Na,K-ATPase pump is macropinocytosed and translocated to MVBs, and accumulating in lysosomes. The Wnt pathway is at the core of many human cancers. 85% of CRCs are initiated by loss-of-function mutations in Adenomatous Polyposis Coli (APC), which increases levels of the transcriptional regulator β-catenin (Kinzler and Vogelstein, 1996; Galluzzi et al., 2019). It was found that the sequestration of the protein complex APC (Axin1, Casein Kinase I alpha), together with GSK3β into multivesicular bodies (MVBs), allows β-catenin to escape proteasomal degradation, leading to nuclear accumulation and β-catenin target gene activation including genes involved in cell proliferation and cell migration (Clevers and Nusse, 2012; Niehrs, 2012; Albrecht et al., 2021). The decrease of GSK3 in the cytosol results in the stabilization of many other GSK3 substrate proteins (some of them are macropinocytosis activators such as Pak1, Ras, and CDC42) in a new signaling field that is known as Wnt stabilization of proteins (Wnt-STOP) (Taelman et al., 2010; Albrecht et al., 2020). Wnt-STOP could play an essential role in the stabilization of the Na,K-ATPase. Activation of the Wnt pathway leads to increased numbers of lysosomes and MVBs.

It has been reported that many cancer cells increase the number of lysosomes and autolysosomes to maintain homeostasis by increasing the degradation and recycling of macromolecules to maintain cell proliferation in order to survive stressful conditions (Kroemer and Jäättelä, 2005; Cardone et al., 2005; Zhitomirsky and Assaraf, 2016). The presence of increased Na,K-ATPase expression and lysosomes could serve as an early marker for colon cancer detection.

The *Xenopus* embryo provides a favorable system for dissecting the molecular mechanisms that control Wnt signaling in development. Early development and tumorigenesis are similar in terms of gene expression, signaling pathways, and cellular behaviors (Ma et al., 2010). Targeting the Wnt pathway is limited by its complexity and involvement in many biological processes. Recent findings have uncovered important links between Wnt signal transduction and membrane trafficking (Albrecht et al., 2021). It was discovered that the dorsalization phenotype by LiCl is blocked by the macropinocytosis inhibitor EIPA (Tejeda-Muñoz and De Robertis, 2022a; Tejeda-Muñoz et al., 2023; Azbazdar et al., 2023), suggesting that dorsal development requires macropinocytosis. Lysosomal inhibition during early development with the V-ATPase inhibitors Bafilomycin, or Concanamycin A (Tejeda-Muñoz and De Robertis, 2022a), and in the present study with Ouabain, highlights membrane trafficking as an emerging critical target for Wnt inhibition. Inhibitors of macropinocytosis or lysosomes can inhibit Wnt signaling; the opposite effect can be achieved by using Phorbol 12-myristate 13-acetate (PMA) or the second messenger lipid diacylglycerol (DAG), both of which can enhance Wnt signaling (Tejeda-Muñoz et al., 2023; Azbazdar et al., 2023). The latter observations may have important implications in understanding the activity of tumor promoters which have a powerful role in cancer progression without directly mutating DNA (Hanahan and Weinberg, 2011).

**Fig. 6.**
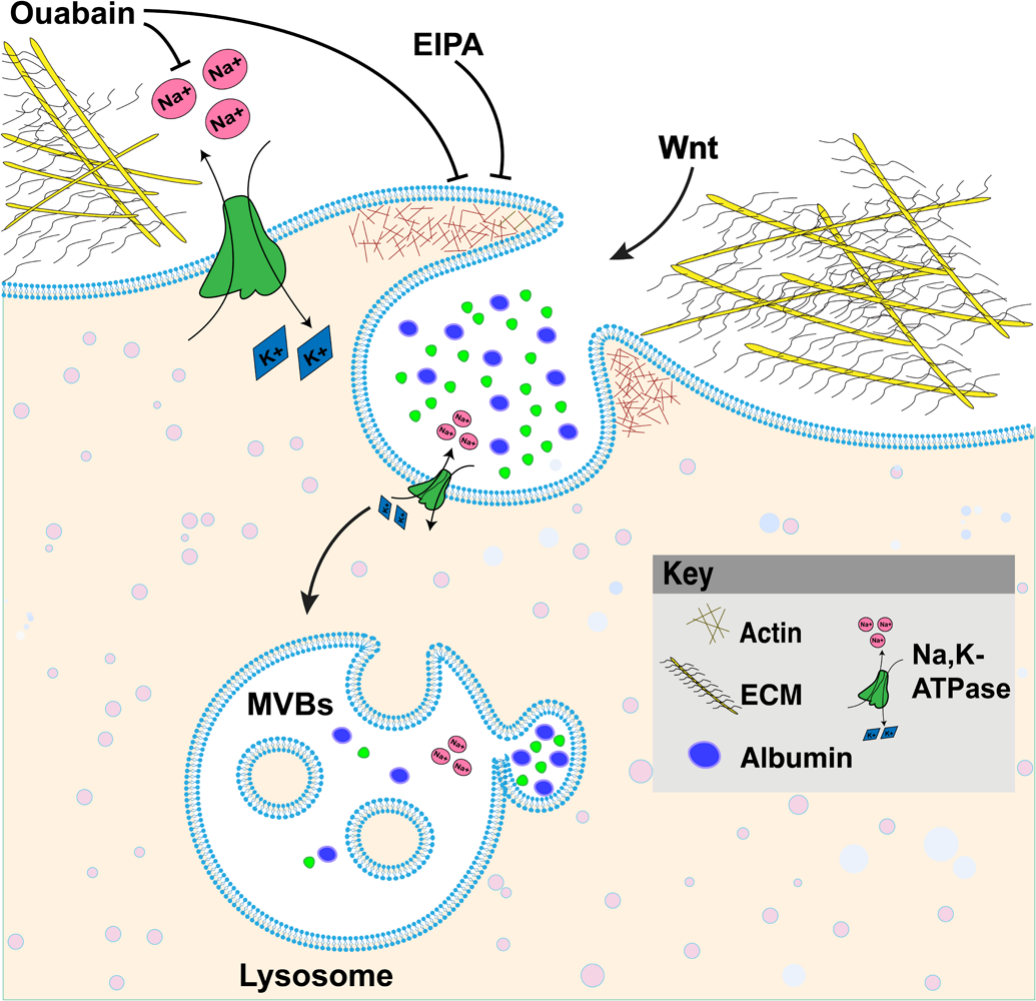
**Model of Ouabain blocking the Na,K-ATPase pump and macropinocytosis, resulting in inhibition of Wnt signaling.** The Na,K-ATPase transmembrane protein is a member of the P-type ATPase family that pumps three Na^+^ ions out of cells while pumping two K^+^ ions into cells, establishing the electrical potential across membranes. The cardiotonic steroid Ouabain is a selective Na,K-ATPase inhibitor targeting the α1-subunit that blocks macropinocytosis, lysosomes, and Wnt signaling.

Ouabain-induced internalization of the Na,K-ATPase into endosomes has been reported in different types of cells (Liu et al., 2004; Yan et al., 2012). Cardiac glycoside-induced endosomal recycling could further activate the degradation of other proteins, thus perturbing cancer cell homeostasis Additional extracellular stimuli such as dopamine (Chibalin et al., 1997), parathyroid hormone (Zhang et al., 1999), hypoxia, lung injury (Dada et al., 2003; Lecuona et al., 2009), hypercapnia (Welch et al., 2010), and sepsis (Berger et al., 2011) have been reported to induce internalization of the Na,K-ATPase. The time course varies from minutes to hours, and the suggested mechanisms involve activation through phosphorylation of α1 by protein kinases and in some cases its subsequent ubiquitination. Na,K-ATPase has been implicated in the EGFR/Src–Ras–ERK pathway and the PI3K1A–PDK–Akt pathway (Lingrel, 2010; Wu et al., 2013). Here, we discovered a new regulation model for Na,K-ATPase, whereby the initiation of Wnt signaling triggers the macropinocytosis of Na,K-ATPase in lysosomes, promoting tumorigenesis (Fig. 6). Membrane trafficking provides new insights (Colozza et al., 2020) that are of possible clinical relevance to our understanding of the role of Na,K-ATPase in Wnt-driven cancers.

## MATERIALS AND METHODS

### Key resources table

**Table:**
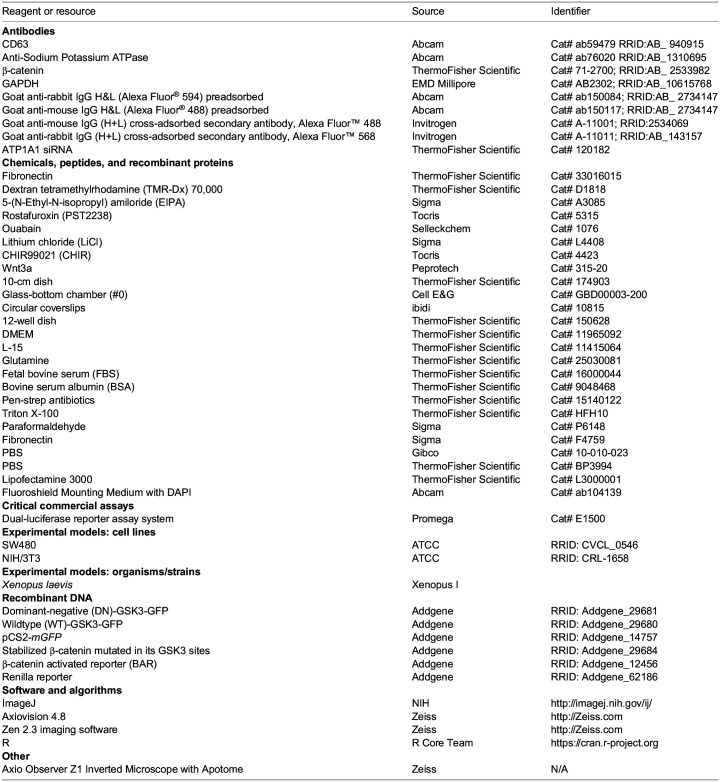


### Data availability

Further information and requests for resources and reagents should be directed to and will be fulfilled by the corresponding author.

No custom code, software, or algorithm central to supporting the main claims of the paper were generated in this manuscript.

### Experimental model and subject details

#### Tissue culture and transfection

HeLa (ATCC, CRL-2648), 3T3, and HEK293T cells stably expressing BAR and Renilla reporters were cultured in DMEM (Dulbecco's Modified Eagle Medium CAT:11965092, Gibco), supplemented with 10% fetal bovine serum (FBS), 1% glutamine, and penicillin/streptomycin. SW480 cells (Faux et al., 2004) were cultured in DMEM/F12 (DMEM: Nutrient Mixture F-12 CAT:11875093, Gibco), supplemented with 5% FBS, 1% glutamine, and penicillin/streptomycin. The cells were seeded at a cell density of 20 to 30%, and experiments were performed when cells reached between 70 to 80% confluency. For transfection, lipofectamine 3000 was used following the instructions from the provider. Cells were seeded in a 12-well plate or chamber and then cells were transfected with the corresponding plasmid. Cells were cultured for 6 to 8 h in a medium containing 2% FBS before all treatments.

#### Ouabain incubation and *in situ* hybridization in *Xenopus* embryo

All animal experiments were approved by the UCLA Animal Research Committee*. Xenopus laevis* embryos were fertilized *in vitro* using excised testis and staged as described (Tejeda-Muñoz and De Robertis, 2022a; Colozza and De Robertis, 2014). *Xenopus* embryos at the 32-cell stage were incubated with Ouabain (10 μM in 20% L-15 culture medium) for 7 min in 0.1 MMR solution. After incubation, embryos were washed twice with 0.1 MMR solution and cultured overnight until the early tailbud tadpole stage. *In situ* hybridizations were performed as described at www.hhmi.ucla.edu/derobertis using *sox2*, *otx2*, and *rx2a* probes.

#### Ouabain and subsequent LiCl incubation in *Xenopus* embryo

Whole embryos at the 32-cell stage were incubated with Ouabain first for 7 min (10 μM in 20% L-15 culture medium). After that, embryos were quickly washed twice with 0.1×MMR solution and subsequently incubated for an extra 7 min in 0.1×MMRsolution containing LiCl (300 mM in 0.1×MMR solution). After the treatment, the embryos were washed two times with 0.1 MMR solution and cultured until the tadpole stage.

#### LiCl and subsequent Ouabain incubation in *Xenopus* embryo

Whole embryos at the 32-cell stage were incubated with LiCl first for 7 min at 300 mM in 0.1×MMR solution. After that, embryos were quickly washed twice with 0.1×MMR solution and subsequently incubated for an extra 7 min with Ouabain (10 μM in 20% L-15 culture medium). After the treatment, the embryos were washed twice with 0.1 MMR solution and cultured until the tadpole stage.

#### Human colon cancer tissue array and immunochemistry

Tissue microarray immunohistochemical was performed as described in (Tejeda-Muñoz et al., 2023). Briefly, three sets of colon cancer tissue arrays (containing 90 cases of adenocarcinoma and 90 adjacent normal colon tissue from TissueArray.com) were double stained in the samples that were deparaffinized in xylene and rehydrated using graded alcohols. For antigen retrieval, slides were incubated at 95°C for 40 min in citrate buffer (10 mM, 0.05% Tween 20, pH 6.0). Tissue sections were then fixed with 4% paraformaldehyde (Sigma #P6148) for 15 min, treated with 0.2% Triton X-100 in phosphate-buffered saline (PBS; Gibco) for 10 min, and blocked with 5% BSA in PBS overnight. Primary and secondary antibodies were added overnight at 4°C. The samples were washed three times with PBS after each treatment, and coverslips were mounted with Fluoroshield Mounting Medium with DAPI (ab104139). Immunofluorescence was analyzed and photographed using a Zeiss Imager Z.1 microscope with Apotome.

#### Antibodies and reagents

Total β-catenin antibody (1:1000) was purchased from Invitrogen (712,700, 1:1000) was obtained from Cell Signaling Technologies. CD63 antibody was obtained from Abcam (ab59479). Antibodies against Sodium Potassium ATPase antibody (ab76020, 1:1000), and secondary antibodies for immunostaining for cells (ab150084, ab150117) (1:300) were obtained from Abcam. Secondary antibodies for immunostaining arrays (A-11001, A-11011 were obtained from Invitrogen). Ouabain (1076) was purchased from Selleckchem. TMR-dextran 70 kDa was obtained from ThermoFisher (D1818).

### Immunostaining and western blotting

HeLa, HEK293T, 3T3, and SW480 cells were plated on glass coverslips and transferred to 2% FBS 6 to 12 h before overnight experimental treatments. Coverslips were acid-washed and treated with Fibronectin (10 µg/ml for 30 min at 37°C, Sigma F4759) to facilitate cell spreading and adhesion. Cells were fixed with 4% paraformaldehyde (Sigma #P6148) for 15 min, permeabilized with 0.2% Triton X-100 in phosphate-buffered saline (PBS; Gibco) for 10 min, and blocked with 5% BSA in PBS for 1 h. Primary antibodies were added overnight at 4°C. Cells were washed three times with PBS, and secondary antibodies were applied for 1 h at room temperature. After three additional washes with PBS, the coverslips were mounted with Fluoroshield Mounting Medium with DAPI (ab104139). Immunofluorescence was analyzed and photographed using a Zeiss Imager Z.1 microscope with Apotome. For Western blotting, cells were lysed with RIPA buffer containing 0.1% Nonidet P-40, 20 mMTris/HCl pH 7.5, 10% glycerol, together with protease (Roche, 04693132001) and phosphatase inhibitors (Calbiochem, 524629).

### Luciferase assay

HEK293T cells stably expressing BAR and Renilla reporters (Albrecht et al., 2020), treated with LiCl (40 mM) or Wnt3a (100 ng/ml), with or without Ouabain (10 µM), were incubated for 8 h. Luciferase activity was measured with the Dual-Luciferase Reporter Assay System (Promega) according to the manufacturer's instructions, using the Glomax Luminometer (Promega). Luciferase values of each sample were normalized for Renilla activity.

### siRNA assay

The siRNA experiment in Fig. S2B was conducted as outlined in reference (8). Briefly, 2 million SW480 cells were utilized due to their constitutively active Wnt signaling. The cells were plated in six-well culture dishes. The following day, knockdown was performed using either siRNA targeting ATP1A1 or scrambled sequences, employing Lipofectamine 3000 transfection reagent in triplicate. On the third day, cells were re-plated onto 12-well plates. On the fourth day, cells were transfected with BAR-Luc Wnt reporter DNA. To normalize transfection efficiency, pCS2+ Renilla was co-transfected, with each well receiving 1.6 μg of DNA (1.2 μg BAR-Luc reporter, 0.4 μg pCS2+ Renilla). The luciferase reporter activity in the cell lysates was measured after 24 h using a Dual-Luciferase assay kit (Promega, Madison, WI, USA).

### Flow cytometry

Hela cells were cultured in a sterile-filtered DMEM medium containing 10% v/v FBS and 1% v/v Penicillin-Streptomycin. Cells were seeded at a density of 1×10^5/well in a 12-well plate and incubated for 20 h, followed by a FBS deprived condition at 2% v/v FBS for 4 h before returning to normal FBS concentration at 10% v/v. Lipofectamine™ 3000 was used as the gene transfection reagent per the manufacturer's instruction. A total of 1 µg PCS2+ATP1A1 (the human gene ATP1A1 was subcloned into PCS2 by GenScript) was added to the respective well. After 24 h of transfection, the medium containing Lipofectamine was removed, and a fresh medium was added. At this point, cells were divided into four groups: group 1: cells without gene transfection; group 2: cells treated with 40 mM LiCl without gene transfection; group 3: cells with gene transfection; and group 4: cells with gene transfection treated with 40 mM LiCl. The cells were further incubated for 24 h. The cell culture medium was removed after incubation. Cells were trypsinized, collected, fixed (4% paraformaldehyde for 15 min), permeabilized (0.1% triton for 10 min), and blocked (5% BSA for 60 min) before immunostaining. For each sample, immunostaining was carried out using a rabbit anti-human beta-catenin polyclonal primary antibody (clone: CAT-15) for 1 h (antibody: 0.25 µg/test), followed by incubation with goat anti-rabbit IgG (H+L) cross-adsorbed secondary antibody conjugated with Alexa Fluor™ 488 for 1 h in the dark (antibody: 0.25 µg/test). Two DPBS washes were performed between each antibody staining via centrifugation at 3000 rpm for 5 min. Stained cells were analyzed using the Accuri™ C6 Plus Flow Cytometer System (BD). Data were processed using FCS Express 7 Flow Cytometry Software (De Novo Software).

### Three-dimensional spheroid cell culture

SW480 cells were cultured in a petri dish using DMEM: F-12 medium with 5% FBS. The top cover was removed from 60 mm tissue culture dishes, and 3 ml of PBS was placed in the bottom of the dish to act as a hydration chamber. Cells were counted, and 500 cells were added as 25 μl drops deposited onto the Petri dish cover and immediately inverted over the humid chamber. Six drops per condition were plated, keeping enough distance between each of them. The inverted drop cultures were incubated at 37°C in 5% CO_2_ and 95% humidity. The drops were monitored daily; after 4 days, aggregates had been formed and Ouabain treatment was added to the spheroids. After 4 days, spheroids were incubated with TMR-dextran 70 kDa (1 mg/ml) for 1 h, and each spheroid was photographed with an Axio Zoom.V16 Stereo Zoom Zeiss microscope with apotome function.

### Time-lapse imaging

For live-cell analyses (as seen in Movie 1), plate the cells on fibronectin in a glass-bottom chamber (#0 cover glass, Cell E&G: GBD00003-200) for 12–18 h. Images were collected in a green fluorescence filter, acquired every 30 s, and acquired using the Zeiss Observer.Z.1 inverted microscope with Apotome.2. For more details, see (Tejeda-Muñoz et al., 2022a).

### Cell viability and proliferation assays

SW480 cells were seeded at 70–80% confluency into a six-well plate. After 24 h the cells were treated overnight with the Ouabain inhibitor. Cells were trypsinized and collected to be measured for cell viability and proliferation with the Vi-CELL XR cell viability analyzer.

### ATP1A1 RNA expression analysis

ATP1A1 RNA expression in normal and adenocarcinoma colon samples was downloaded from the Human Protein Atlas and analyzed using the DESeq2 package via the coding program R-Studio (Love et al., 2014). Bean plots in Fig. 1 were generated using fragments per kilobase of exon per million mapped fragments (FPKM) values as input using the BoxPlotR package (Spitzer et al., 2014). For reproducibility purposes, colon adenocarcinoma datasets were screened to include only samples acquired through the Cancer Genome Atlas (TCGA) program (Tomczak et al., 2015). Both normal and adenocarcinoma colon datasets possessed an approximate male-to-female ratio of 1:1. ATP1A1 expression data was extracted from 373 normal tissues and 596 adenocarcinoma samples. Of the 596 adenocarcinoma samples, 471 (79%) were between stages I and II, and 174 (29%) were at stage III or more advanced. ATP1A1 expression in normal colon and adenocarcinoma samples were analyzed for significance in Excel via a two-tailed heteroscedastic Student's *t*-test.

### qRT-PCR

Quantitative RT-PCR experiments using *Xenopus* embryos were performed as previously described (Colozza and De Robertis, 2014). Primer sequences for qRT-PCR were as follows:

**Table:**
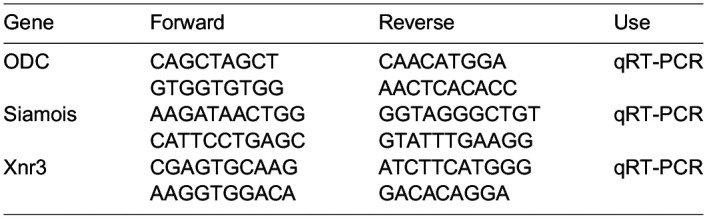


### Quantification and statistical analysis

Data were expressed as mean and s.e.m. Statistical data analysis was performed using the Student’s *t*-test; a *P*-value of <0.01** was considered statistically significant for differences between means. Fluorescence was quantified in control versus treated cells using ImageJ software analyses with *n*>30 cells or *n*>30 images from arrays of human samples per condition. Fluorescence intensity was normalized in images compared in each condition, and results from three or more independent experiments were presented as the mean±s.e.m.

## Supplementary Material

10.1242/biolopen.060269_sup1 Supplementary information
