# Supplementary material for: Na,K-ATPase activity promotes macropinocytosis in colon cancer via Wnt signaling
Source: Biol Open. 2024 May 21;13(5):bio060269. doi: 10.1242/bio.060269 (PMC11139033; doi:10.1242/bio.060269)
Supplement: Supplementary information [file biolopen-13-060269-s1.pdf]

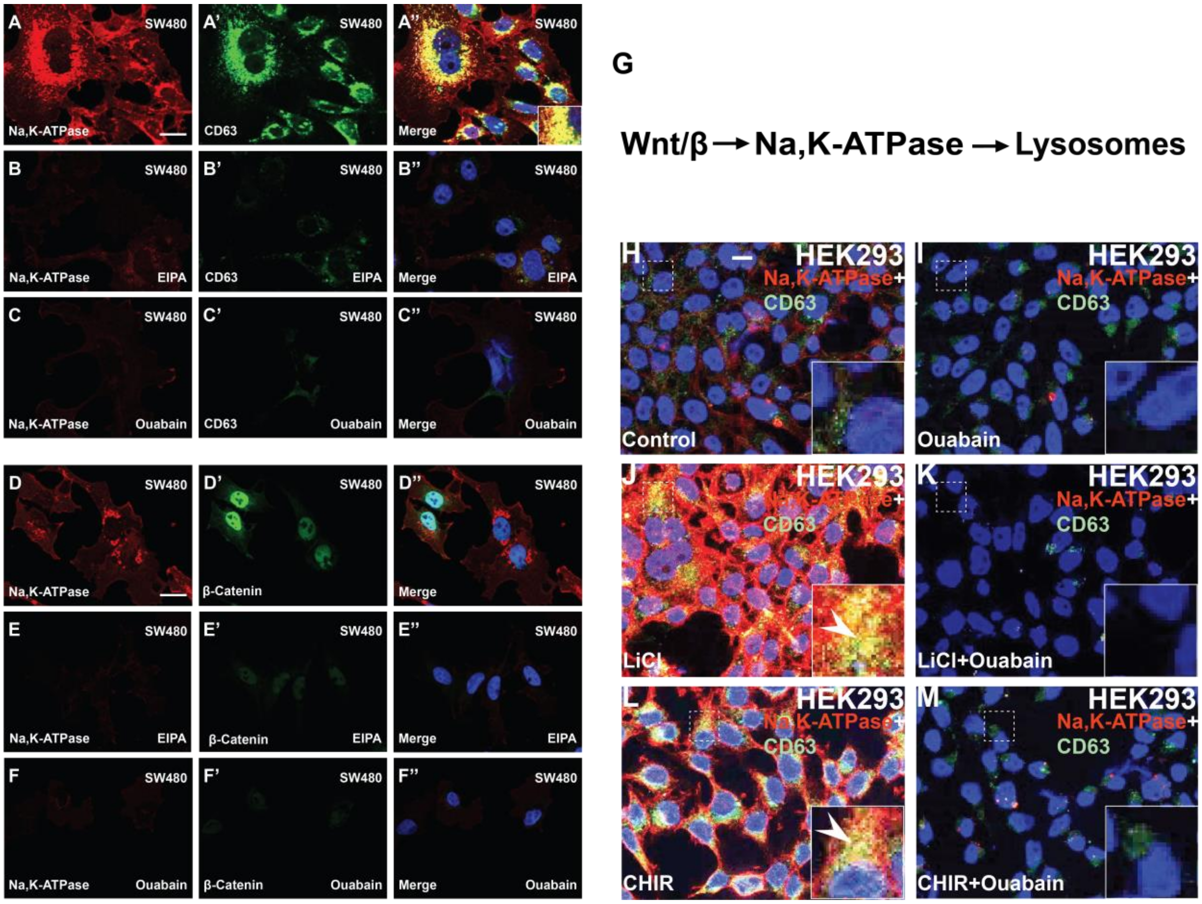

Fig. S1. The Na,K-ATPase is Accumulated in Lysosomes by Wnt Pathway Activation and Depends on Macropinocytosis; Related to Figure 2

(A-A'') The human SW480 colon adenocarcinoma cell line, which constitutive Wnt, was stained with Na,K-ATPase and CD63 antibodies, which colocalized.

(B-B'') SW480 cells treated with the EIPA (40 μM) macropinocytosis inhibitor have reduced levels of Na,K-ATPase, and CD63.

(C-C'') The Na,K-ATPase inhibitor Ouabain (200 nM) reduces CD63 levels, presumably through the inhibition of macropinocytosis.

(D-D'') SW480 cells stained with Na,K-ATPase and β-catenin antibodies.

(E-E'') The macropinocytosis inhibitor EIPA reduces Na,K-ATPase levels and blocks Wnt signaling.

(F-F'') Ouabain decreases Na,K-ATPase and β-catenin accumulation.

(G) Diagram illustrating that activation of Wnt causes the endocytosis of the Na,K-ATPase in lysosomes.

(H) Control HEK293 cells stained with CD63 and Na,K-ATPase antibodies.

(I) Ouabain (1 µM) overnight treatment reduced basal levels of Na,K-ATPase.

(J) The activation of the Wnt pathway via GSK3 inhibition with LiCl (40 mM) strongly increased Na,K-ATPase and CD63 levels.

(K) Ouabain treatment strongly reduced the levels of CD63 and Na,K-ATPase that were enhanced by LiCl.

(L) A second GSK3 inhibitor CHIR99021 (8 nM) was used to validate the increased levels of CD63 and Na,K-ATPase caused by GSK3 inhibition (which is a potent inducer of macropinocytosis).

(M) Ouabain blocked the stabilization of Na,K-ATPase (and CD63) induced by CHIR99021.

Data from four independent experiments; Scale bars 10 µm.

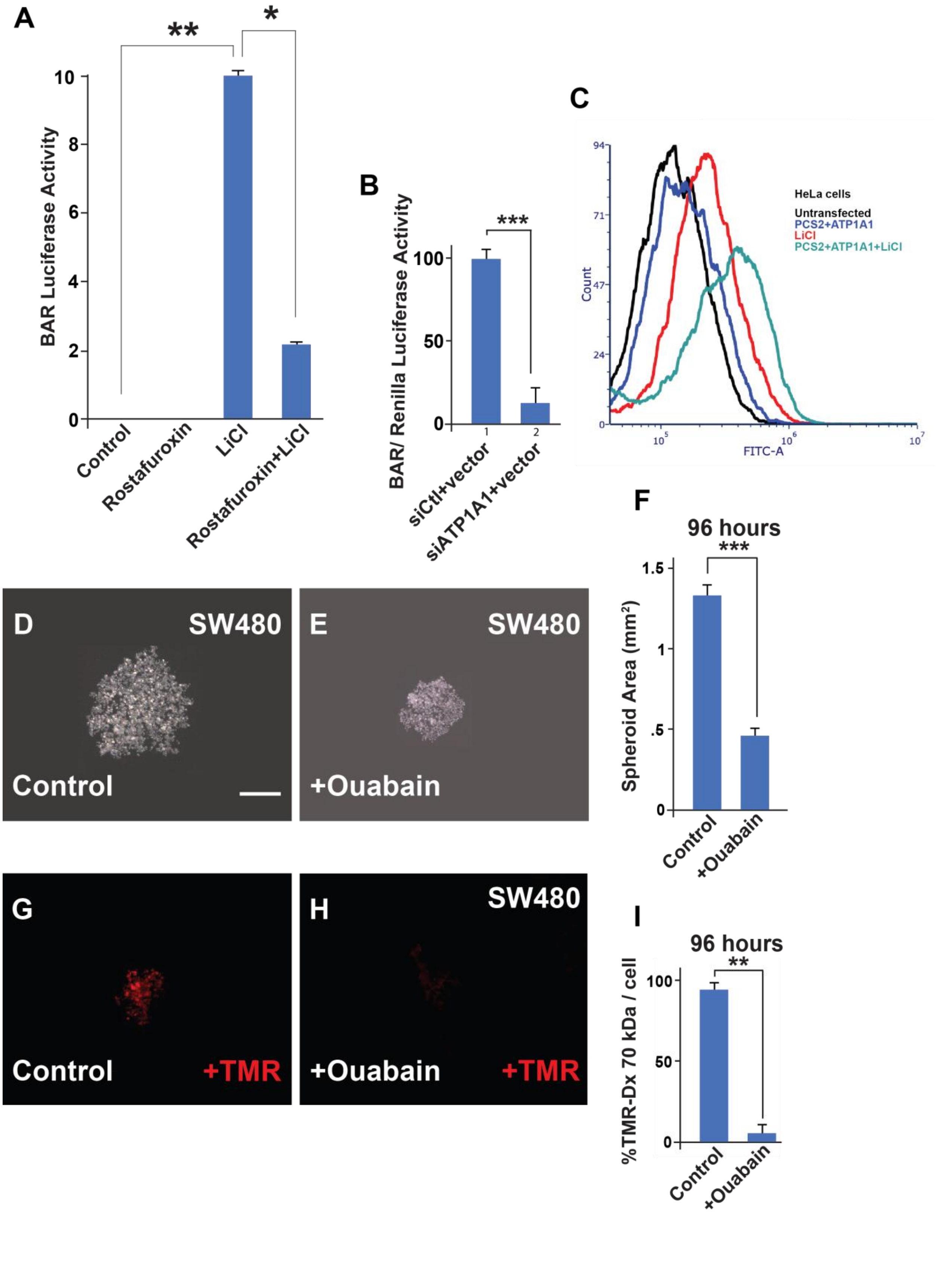

Biology Open • Supplementary information

Fig. S2. Ouabain Blocks Macropinocytosis in CRC SW480 Spheroids; Related to Figure 3

(A) Rostafuroxin/PST2238, which binds specifically to the extracellular domain of ATP1A1, used as a second Na,K-ATPase inhibitor, decreased β-catenin signaling stimulated by LiCl.

(B) The depletion of ATP1A1 (A1-subunit of the NA,K-ATPase pump) using a siRNA in SW480 cells. This results in decreased β-catenin transcriptional activity as measured by the BAR-luciferase assay.

(C) Flow cytometry analysis shows that overexpression of ATP1A1 increases β-catenin levels in HeLa cells. Black: untransfected control HeLa cells; blue: HeLa cells overexpressing ATP1A1; red: untransfected HeLa cells treated with LiCl; green: HeLa cells overexpressing ATP1A1 and treated with LiCl. Each experiment was conducted three or more times. The overexpression of ATP1A1 (blue) shows no significant changes in β-catenin levels compared to controls. The treatment by LiCl (red) increases the β-catenin level 1.7-fold compared to controls (unpaired t-test, $t = 8.437$, $df = 4$, $**p = 0.0011$). The overexpression ATP1A1 combined with LiCl treatment (green) increases the β-catenin level 2.2-fold compared to controls (Unpaired t-test, $t = 8.563$, $df = 4$, $**p = 0.0010$) and 1.3-fold compared to LiCl treatment only (Unpaired t-test, $t = 3.429$, $df = 4$, $*p = 0.0266$). Thus, we conclude that the overexpression of ATP1A1 enhances the activation of the Wnt pathway.

(D) SW480 spheroids after 96 hours in inverted drop culture.

(E) Ouabain (40 μM) treatment affects the ability to form spheroids in 3D cultures.

(F) Quantification of the spheroid area after Ouabain treatment.

(G) Spheroids incubated with the macropinocytosis marker TMR-dextran 70kDa (1 mg/mL) for 1 hour.

(H) Ouabain treatment reduces macropinocytosis of TMR-dextran 70 kDa.

(I) Quantification of macropinocytic uptake inhibition caused Ouabain treatment.

All experiments with cultured cells were biological triplicates.

Eight spheroids were plated per condition, in triplicate.

Error bars denote SEM ($n \geq 3$) ($***\ p < 0.001$, $**\ p < 0.01$, and $*\ p < 0.05$). Scale bars, 500 μm.

Biology Open • Supplementary information

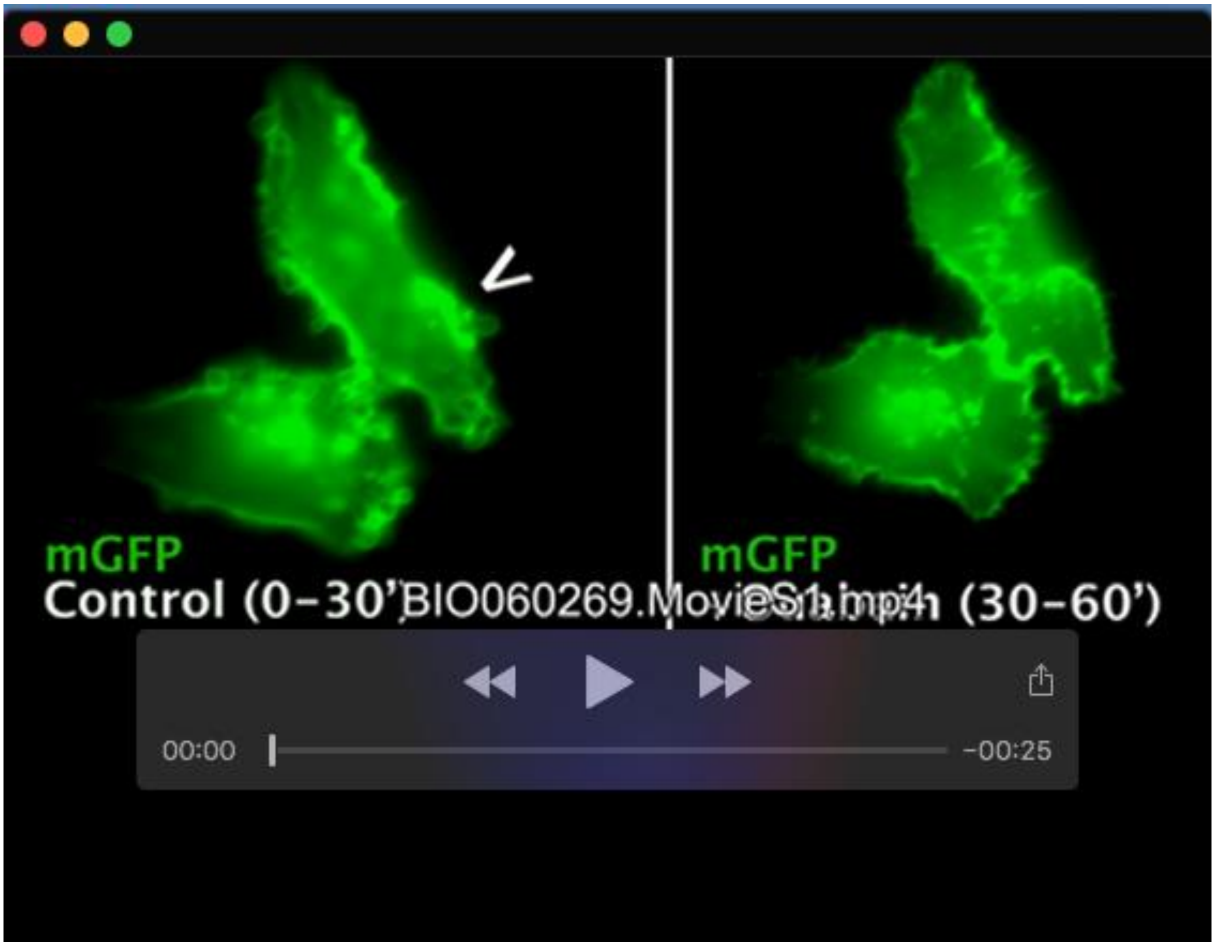

Movie 1. The Sustained Plasma Membrane Macropinosome Ruffles Characteristic of Cancer Cells with Activating Wnt Pathway Mutation Stop within Minutes of Addition of Ouabain; Related to Fig. 3

SW480 cells have sustained macropinocytosis [5] that involves the uptake of significant extracellular fluid and solutes by forming large vesicles (> 0.2 µm) called macropinosomes (arrow) that were reduced rapidly after treatment with Ouabain (200 nM). Since macropinocytosis is required for Wnt signaling [50]-[54], blocking macropinocytosis explains the potent inhibitory effects of Ouabain on canonical Wnt signaling.
